# Green-Lyte: A Multidisciplinary Quality Improvement Project to Optimize Electrolyte Replacement and Reduce Environmental Burden in ICUs at a Comprehensive Cancer Center

**DOI:** 10.1097/CCE.0000000000001508

**Published:** 2026-11-12

**Authors:** Lina Khashashneh, Asma Kharabsheh, Naser Mahmoud, Musab Smadi, Tala Abdel-Haq, Lujain Alessa, Mohammed Al-Jaghbeer, Wedad Awad, Naheel Said, Armen Mousa, Lama Nazer

**Affiliations:** 1 Department of Pharmacy, King Hussein Cancer Center, Amman, Jordan.; 2 Department of Medicine, King Hussein Cancer Center, Amman, Jordan.; 3 Nursing Department, King Hussein Cancer Center, Amman, Jordan.

**Keywords:** carbon footprint, electrolytes, environment, intensive care units, quality improvement, waste management

## Abstract

**BACKGROUND:**

Electrolyte replacement therapies are commonly prescribed in ICUs. However, studies have shown that electrolytes are frequently administered to patients with no clear clinical indications and, in most cases, the IV route is used. In this study, we evaluated the impact of a multidisciplinary quality improvement project, Green-Lyte, on electrolyte replacement and on the environmental burden.

**METHODS:**

This was a quality improvement retrospective study conducted in the adult ICUs at a comprehensive cancer center comparing preimplementation (September 2024 to February 2025) and postimplementation (May 2025 to October 2025) phases of the Green-Lyte project. Green-Lyte consisted of defined criteria to guide both the indication and route of administration for potassium, magnesium, and phosphorus replacement, along with ongoing education and follow-up. Outcomes included the number of electrolyte prescriptions, concordance with Green-Lyte criteria for indication and route of administration, as well as the generated waste and estimated material-associated carbon footprint. Outcomes were standardized per 100 ICU patient-days.

**RESULTS:**

Over the study period, a total of 603 IV and oral electrolyte replacement doses were prescribed in the pre-Green-Lyte phase, and 573 IV and oral electrolyte replacement doses were prescribed in the post-Green-Lyte phase. When adjusted per 100 ICU patient-days, 30.7 doses vs. 19.4 doses were dispensed in the pre- and postimplementation phases, respectively, representing a 36.9% reduction in prescribed electrolytes. Concordance with the Green-Lyte criteria for indication increased from 57.8% to 72.8%, representing a 26% relative improvement, whereas concordance with the criteria for route of administration increased from 22.5% to 45.9%, representing a 104% relative improvement. In addition, the generated waste and the estimated material-associated carbon footprint decreased by 61.8% and 63.2%, respectively.

**CONCLUSIONS:**

The Green-Lyte project was associated with decreased utilization of electrolytes and reduced environmental burden, as demonstrated by decreases in generated waste and estimated material-associated carbon footprint.

**KEY POINTS**

**Question:** What is the impact of the Green-Lyte, a multidisciplinary quality improvement project, on electrolyte management in critically ill patients and the environmental burden?

**Findings:** Following implementation of Green-Lyte, electrolyte prescribing decreased by 36.9%, with a relative improvement in prescribed indications and routes of administration by 26% and 104%, respectively. In addition, the generated waste and material-associated carbon footprint decreased by 61.8% and 63.2%, respectively.

**Meaning:** A multidisciplinary quality improvement project to optimize electrolyte management in critically ill patients can reduce electrolyte utilization, improve prescribing practice, and decrease environmental burden.

The climate crisis is recognized as a major threat to human health, and healthcare systems are significant contributors to greenhouse gas emissions. Recent data reports from multinational studies showed that healthcare systems account for a mean of 4–5% of total emissions, with a substantial proportion arising from supply chain activities, including the production and distribution of pharmaceuticals and medical products (1–3). Therefore, healthcare institutions are recognized as important settings for climate mitigation and sustainability quality improvement initiatives.

Within hospitals, ICUs are associated with higher greenhouse gas emissions and waste generation per bed-day than general inpatient wards, producing 2–3 times more CO_2_-equivalent per bed-day than standard wards, with estimates of the environmental impact reaching up to 12 kg CO_2_ equivalent per ICU patient per day (4–6). This high footprint is driven by the ICU’s heavy reliance on single-use items, disposable personal protective equipment, and organ support interventions such as continuous renal replacement therapy and mechanical ventilation, as well as the high medication usage due to critical illness, as critically ill patients were reported to receive twice the number of medications compared with non-ICU patients (5, 7–9).

Electrolyte replacement is one of the most common interventions in ICUs (10). However, studies have shown that electrolytes are frequently administered to patients with serum levels within the normal range, suggesting potential overuse without clear clinical indications (9–11). Furthermore, although oral replacement can be effective for selected electrolyte abnormalities, IV formulations remain the dominant ICU practice.

Recently, the Society of Critical Care Medicine Sustainability Task Force published a report outlining actionable strategies to support environmental stewardship in ICUs (12). Medication prescribing was identified as a key area for reducing the environmental impact of critical care, with recommendations emphasizing medication stewardship, including ensuring appropriate indications for prescribed therapies and converting IV medications to oral formulations when clinically appropriate.

In alignment with our ongoing efforts to optimize medication use and promote environmental sustainability, we implemented the Green-Lyte, a multidisciplinary quality improvement project focused on “Green electro-Lyte replacement.” The project aimed to optimize electrolyte replacement practices among critically ill patients and reduce their environmental impact. In this report, we describe the Green-Lyte project and evaluate its impact on electrolyte prescribing and environmental burden in ICUs at a Comprehensive Cancer Center.

## MATERIALS AND METHODS

### Study Design and Setting

This was a pre- and postquality improvement study with retrospective data collection conducted at King Hussein Cancer Center (KHCC), a 360-bed comprehensive cancer teaching hospital in Amman, Jordan. KHCC has two adult medical-surgical ICUs, with a total of 21 beds, that operate as closed units with high-intensity staffing in accordance with the Leapfrog standards, as previously described (13). The ICUs provide care for patients with cancer presenting with oncology- and non-oncology-related medical and surgical critical illnesses. Surgical cases are primarily those related to cancer-related surgeries and do not include cardiac, trauma, or other specialized types of surgeries. The characteristics of patients admitted to the ICUs, type of admissions, and outcomes have been described in a previous publication (14).

This quality improvement study is reported in accordance with the Standards for Quality Improvement Reporting Excellence (SQUIRE) 2.0 guidelines (15). A completed SQUIRE checklist is provided in **Appendix 1** (https://links.lww.com/CCX/B703). The study included two phases: a 6-month preimplementation phase (September 2024 to February 2025) and a 6-month postimplementation phase (May 2025 to October 2025).

The study was conducted in accordance with the ethical standards of the KHCC Institutional Review Board (IRB) and with the Declaration of Helsinki 1975. It was approved by the KHCC IRB on July 8, 2025 (IRB# 25KHCC182), under the study title: Green-Lyte-Optimizing electrolyte therapy: retrospective evaluation of electrolyte replacement in adult ICU patients. A waiver of informed consent was granted due to the retrospective nature of the study.

### Patient Population

The study included adult patients (≥ 18 yr old) treated in the ICUs during the study period and who received electrolyte replacement therapy for potassium, magnesium, or phosphorus during their stay. Electrolyte replacement refers to oral and IV doses of electrolyte supplementation. Electrolytes that were added as part of the maintenance IV fluids were excluded.

### Quality Improvement Project Green-Lyte

The Green-Lyte was a multidisciplinary quality improvement project that defined criteria to guide both the indication and route of administration for potassium, magnesium, and phosphorus replacement. The Green-Lyte criteria were developed by a multidisciplinary team that consisted of critical care pharmacists, physicians, and nurses.

An indication for electrolyte replacement was considered “concordant with Green-Lyte criteria” if the patient had documented low serum concentrations before the administration of the relevant electrolyte or had serum concentrations within the normal range but with a documented clinical indication for replacement, such as severe diarrhea or continuous renal replacement therapy. Normal electrolyte ranges were defined according to the institutional laboratory reference values. To ensure that the Green-Lyte project accounted for cases in which patients may require proactive electrolyte replacement despite normal levels, we did not specify cases eligible for deviation, provided that the prescribing physician provided a clinical rationale.

As for the route of administration, the oral route was considered the preferred option in patients receiving enteral nutrition or prescribed at least one oral medication. The IV route was considered concordant with Green-Lyte criteria in patients who did not meet criteria for oral eligibility or when a reason for IV administration was documented, despite oral eligibility, such as vomiting and diarrhea. Similar to the indication criteria, we did not specify the clinical conditions in which the oral route would not be suitable for patients. Instead, this decision was left to the prescribing physician, provided that the clinical rationale for using the IV route was documented.

The order entry and processing workflow of electrolyte replacement remained unchanged before and after implementation of the Green-Lyte initiative. In our ICUs, electrolyte replacement orders are entered electronically as patient-specific orders when replacement is clinically indicated; no standardized electrolyte order sets or preemptive “as needed” electrolyte replacement protocols are used at our institution. Physicians enter electrolyte replacement orders electronically, which are then assessed and processed by pharmacists before administration, except in critical emergencies requiring immediate treatment. The prescribing physicians are typically the ICU physicians and the critical care fellows who cover our ICUs.

Following implementation of the Green-Lyte initiative, pharmacists assessed each order for compliance with the Green-Lyte criteria, including the indication and route of administration, as outlined in **Appendix 2** (https://links.lww.com/CCX/B703). If an order did not meet the predefined Green-Lyte criteria, the pharmacist contacted the prescribing physician to discuss the order and recommend modifications in accordance with the criteria. When a rationale for deviating from the Green-Lyte criteria was provided, it was documented as a comment in the electronic order; otherwise, the comment field was left blank. Physicians were also encouraged to document their rationale for any deviation directly in the order to minimize the need for pharmacist clarification.

The implementation of Green-Lyte was in April 2025. Approximately 1 month prior to implementation, emails were sent to all ICU staff introducing the project and outlining the Green-Lyte guideline criteria and implementation process. In addition, several educational sessions were conducted, which included physicians, nurses, and pharmacists working in the ICUs. The sessions were in-person and focused on the rationale for the initiative, the Green-Lyte electrolyte replacement criteria, and the proposed changes in clinical practice. Following implementation, all electrolyte replacement orders were assessed weekly during the first month to evaluate compliance with the Green-Lyte criteria. Orders that did not meet one or more criteria were reviewed with the relevant healthcare team members to provide feedback and support practice improvement. After this initial 1-month period, compliance assessments were conducted monthly.

### Data Collection and Study Outcomes

The electronic medical records were used to identify all patients who received IV or oral replacements of potassium, magnesium, or phosphorus during their stay in the ICU. The total number of prescribed oral and IV replacement therapies for each electrolyte was determined during the pre- and post-Green-Lyte study phases. For each prescribed electrolyte replacement, we assessed the indication and route of administration according to the criteria defined by the Green-Lyte. In cases where electrolytes were administered despite normal serum concentrations or where the IV route was used despite eligibility for oral administration, the order was reviewed to determine whether a clinical justification had been documented. If a clinical rationale was provided, the indication or route was considered concordant with Green-Lyte criteria; if no justification was documented, it was classified as non-concordant with the criteria.

The environmental impact of electrolyte replacement was evaluated by assessing the generated waste and estimated material-associated carbon footprint associated with each replacement. In addition, because the preparation of electrolyte formulations requires staff time and effort, we also assessed the time required for preparation of both IV and oral formulations.

The waste generated from IV and oral electrolyte replacement therapies was quantified by weighing all single-use materials associated with the preparation and checking of each electrolyte replacement. For IV formulations, the waste consisted of electrolyte vials/ampoules, syringes, IV solution bags, and infusion line sets. For the oral formulations, the waste consisted of oral unit-dose syringes for the oral electrolyte solutions, dispensing disposable medication bottles, and the disposable prepackaged unit-dose cups for the capsules and tablets. In addition, we accounted for gowns, gloves, and masks used during the preparation of IV formulations. Each component was weighed individually, and the cumulative weights were used to determine the total waste generated for each electrolyte preparation.

The carbon footprint associated with the electrolyte preparation was estimated using published life-cycle assessments for both plastic and glass materials. The reported emission factors for plastic (including polyvinyl chloride) ranged from 2.9 to 4.4 kg CO_2_e per kg, whereas those for glass ranged from 0.57 to 0.89 kg CO_2_e per kg (16–18). For the purposes of this study, fixed emission factors based on the reported values were applied, including 3.179 kg CO_2_e per kg for plastic and 0.733 kg CO_2_e per kg for glass.

The workload associated with the preparation and checking of the electrolytes was determined by measuring the time required to prepare each type of electrolyte formulation. For IV formulations, preparation time included the time required for aseptic compounding in the laminar airflow hood, while for oral formulations, it included the time needed to prepare the oral syringes and tablets or capsules. Time measurements were obtained through direct observation of the preparation of IV and oral potassium, magnesium, and phosphorus. Preparation time was estimated by measuring multiple preparations for each formulation performed by pharmacy assistants and pharmacists with varying levels of experience, and the average time was used in our calculations.

### Statistical Analysis

The study outcomes were compared between the pre-Green-Lyte and post-Green-Lyte phases. Each dose of administered electrolyte was considered an event and was evaluated for concordance with Green-Lyte criteria, as well as associated waste, estimated material-associated carbon footprint, and workload. To account for variability in patient census in the pre- and postphases, electrolyte consumption was standardized per 100 ICU patient-days. The appropriateness of both the indication and the route was analyzed as a proportion of the prescribed electrolytes, and the waste, material-associated carbon footprint, and workload were adjusted per 100 patient-days.

Continuous outcomes were compared using the two-sample equal variance Student *t* test, whereas categorical outcomes were compared using the chi-square test. A *p* value of less than 0.05 was considered statistically significant.

## RESULTS

Over the study period, a total of 603 IV and oral electrolyte replacement doses were prescribed during 112 patient admissions in the pre-Green-Lyte phase and 573 doses were prescribed during 114 patient admissions in the post-Green-Lyte phase. **Table 1** outlines the characteristics of the patient admissions with electrolyte replacements during each phase. The pre-Green-Lyte phase consisted of 2118 ICU patient-days, whereas the post-Green-Lyte phase consisted of 3066 ICU patient-days. Potassium was the most frequently prescribed electrolyte, accounting for 290 doses (48.9%) in the preimplementation phase vs. 381 (64.0%) in the postimplementation phase, followed by magnesium (222 [34.1%] vs. 134 [22.5%]) and phosphorus (91 [14.0%] vs. 80 [13.4%]). In both phases, there were no changes in electrolyte testing practices, as electrolyte measurements were obtained using serum laboratory testing, and point-of-care testing was not used to guide electrolyte replacement decisions. In addition, there were no reported shortages of any IV or oral electrolyte formulations at our institution that may have affected electrolyte utilization.

**TABLE 1.** Characteristics of Patients Who Received Electrolyte Replacements During the Pre- and Post-Green-Lyte Phases

Characteristics	Pre-Green-Lyte (*n* = 112)	Post-Green-Lyte (*n* = 114)
Age, yr, mean ± sd	59.06 ± 19	60.64 ± 11.33
Sex, *n* (%)		
Males	43 (38.4)	61 (53.5)
Females	69 (61.6)	53 (46.5)
Type of malignancy, *n* (%)		
Solid tumors	103 (92)	101 (89)
Hematologic malignancies	9 (8)	13 (11)
Admission diagnosis, *n* (%)		
Sepsis	38 (34)	34 (30)
Respiratory failure	23 (20)	24 (21)
Cardiovascular	20 (18)	22 (19)
Electrolyte imbalance	7 (6)	5 (5)
Neurologic	4 (4)	4 (3)
Others	20 (18)	25 (22)
Mechanical ventilation during ICU stay, *n* (%)	32 (29)	45 (39)
Vasopressors during ICU stay, *n* (%)	30 (27)	41 (36)
ICU length of stay, d, mean (sd)	5.3 (6.7)	4.5 (6.1)
ICU mortality, *n* (%)	34 (30)	37 (32)

When adjusted per 100 ICU patient-days, a total of 30.7 doses vs. 19.4 doses were prescribed in the pre- and postimplementation phases, respectively, representing a 36.9% reduction in prescribed electrolytes. **Table 2** outlines the prescribed doses of electrolytes per 100 ICU patient-days in the pre- and postimplementation phases for each electrolyte and route. For all electrolytes, there was a statistically significant decrease in the IV doses from 28.75 to 13.63 per 100 ICU patient-days (*p* < 0.001) with an increase in the use of the oral route from 1.98 to 5.77 doses per 100 ICU patient-days (*p* < 0.001).

**TABLE 2.** Electrolyte Doses Prescribed in the Pre- and Postimplementation Phases

Electrolyte	Preimplementation Doses Per 100 Patient-Days	Postimplementation Doses Per 100 Patient-Days
All electrolytes	30.74	19.41
Oral	1.98	5.77
IV	28.75	13.63
Potassium	15.96	12.43
Oral	1.42	3.49
IV	14.54	8.94
Magnesium	10.48	4.37
Oral	0.09	1.37
IV	10.39	3.0
Phosphorous	4.30	2.61
Oral	0.47	0.9
IV	3.82	1.7

### Optimizing Electrolyte Management

**Table 3** summarizes concordance with Green-Lyte criteria for electrolyte prescriptions in the pre- and postimplementation phases. Compared with the preimplementation phase, the postimplementation phase was associated with significant improvements across all electrolytes in meeting the Green-Lyte criteria. Overall, the proportion of prescribed electrolytes that met the Green-Lyte criteria for indications increased from 57.8% to 72.8%, reflecting a 26% improvement, whereas the proportion with route of administration that met the criteria increased from 22.5% to 45.9%, reflecting a 104% increase.

**TABLE 3.** Concordance with Green-Lyte Criteria for Electrolyte Prescriptions in the Pre- and Postimplementation Phases

Electrolyte	Preimplementation Criteria-Concordance, *n* (%)	Postimplementation Criteria-Concordance, *n* (%)
All electrolytes	Total = 603	Total = 595
Indication	349 (57.8)	433 (72.8)
Route	136 (22.5)	273 (45.9)
Potassium	Total = 290	Total = 381
Indication	219 (75.5)	305 (80.1)
Route	52 (17.9)	153 (40.2)
Magnesium	Total = 222	Total = 134
Indication	52 (23.4)	61 (45.5)
Route	47 (21.2)	78 (58.2)
Phosphorous	Total = 91	Total = 80
Indication	78 (85.7)	67 (83.7)
Route	37 (40.7)	42 (52.5)

### Environmental Burden

**Table 4** summarizes the generated waste, estimated material-associated carbon footprint, and the preparation time for the administered electrolytes in the pre- and postimplementation phases, adjusted per 100 patient-days. The total waste generation was 17.8 kg per 100 patient-days in the preintervention phase and 6.8 kg per 100 patient-days in the postintervention phase, representing a 61.8% reduction. The estimated total material-associated carbon footprint was 50.6 kg CO_2_e per 100 patient-days and 18.6 kg CO_2_e per 100 patient-days in the pre- and post-Green-Lyte phases, respectively, representing a decrease of 63.2%.

**TABLE 4.** Waste, Carbon Footprint, and Preparation Time for Electrolytes in the Pre- and Postimplementation Phases

Electrolyte	Preimplementation Per 100 Patient-Days	Postimplementation Per 100 Patient-Days
All electrolytes		
Waste^a^	17.8	6.8
Carbon footprint^b^	50.6	18.6
Preparation/checking^c^	3.7	2.3
Potassium		
Waste^a^	9.27	4.27
Carbon footprint^b^	26.5	11.8
Preparation/checking^c^	2	1.6
Magnesium		
Waste^a^	4.58	1.77
Carbon footprint^b^	12.94	4.29
Preparation/checking^c^	1.2	0.4
Phosphorous		
Waste^a^	3.59	0.77
Carbon footprint^b^	11.7	2.44
Preparation/checking^c^	0.5	0.2

a Reported in kilograms.

b Reported in kg CO_2_e.

c Reported in hours.

The total time necessary for pharmacy preparation of electrolytes was also reduced from 3.7 hours per 100 patient-days in the preintervention phase to 2.3 hours per 100 patient-days in the postintervention phase, reflecting a decrease by 37.8%. IV preparation time was 3.5 hours per 100 ICU patient-days in the preintervention phase and 1.7 hours per 100 ICU patient-days in the postintervention phase. Oral preparation time was 0.2 hours per 100 ICU patient-days in the preintervention phase and 0.6 hours per 100 ICU patient-days in the postintervention phase.

## DISCUSSION

In this report, we describe a pre-post study to evaluate Green-Lyte, a multidisciplinary quality improvement project aimed at optimizing electrolyte replacement in critically ill patients. This project was associated with a significant reduction in electrolyte replacement prescribing and an increase in the use of the oral rather than IV route of administration. In addition, improved utilization resulted in reduced waste, a lower material-associated carbon footprint, and decreased pharmacy workload. The project was evaluated for more than 6 months among critically ill patients with cancer treated in a medical/surgical ICU. The Green-Lyte incorporated standardized criteria for the indication and route of administration but also allowed flexibility in situations where the Green-Lyte criteria were deemed not clinically appropriate or feasible. This approach enhances the generalizability of such a project and accounts for real-world clinical practice.

Electrolyte replacement is among the most common interventions in ICUs. However, studies have demonstrated that electrolytes are frequently administered despite serum electrolyte concentrations being within the normal range (8, 11). In a large study by Ghannam et al (11), among 20,375 potassium repletion events, 89.3% of the cases were administered with potassium serum level within the normal range and among 8,118 magnesium repletion events, 81.2% of the cases where magnesium was replaced, the concentration was within the normal range. Furthermore, the effect of replacement on serum electrolyte concentrations was often modest, and the associated clinical benefits remain uncertain (19).

In clinical practice, potassium and magnesium supplementation are considered to help prevent arrhythmias, particularly in patients following cardiac surgery or those considered at high risk for arrhythmias. However, current evidence does not support this rationale. In a study by Lancaster et al (20), potassium and magnesium supplementation were not associated with protection against postoperative atrial fibrillation. In fact, at the time of atrial fibrillation onset, or the matched time point in controls, patients who developed postoperative atrial fibrillation had higher mean potassium (4.30 ± 0.44 vs. 4.21 ± 0.43 mmol/L; *p* = 0.001) and magnesium (2.33 ± 0.39 vs. 2.16 ± 0.29 mg/dL; *p* < 0.001) concentrations compared with controls. Furthermore, Joseph et al (19) examined the relationship between maximum heart rate in the 24 hours before and after potassium or magnesium repletion, which did not result in a decrease in maximum postrepletion heart rate.

Several studies described interventions aimed at improving and optimizing electrolyte replacement in the ICUs (21–26). However, these interventions primarily focused on individual electrolytes or protocol-driven approaches, including nurse-driven or computer-assisted protocols. In contrast, the Green-Lyte incorporated predefined criteria into the routine ordering and medication-processing workflow and addressed the three major electrolytes routinely replaced in the ICU: potassium, magnesium, and phosphorus. Furthermore, in addition to evaluating electrolyte utilization and adherence to the criteria, Green-Lyte assessed environmental outcomes, including waste and carbon footprint, as well as pharmacist workload.

In our study, our initiative relied on the prescribing physician to comply with the criteria or provide a justification for deviation, and it relied on the pharmacist processing the orders to ensure that the orders were compliant with the criteria. However, given that this was dependent on the prescriber and activator, this may have contributed to cases of noncompliance after the implementation. We plan to consider incorporating it into the order entry process and activation process.

Prasad et al (27) described an artificial intelligence reinforcement learning approach to guide efficient, effective, and patient-specific electrolyte replacement in critically ill patients. The model was developed using patient data from the University of Pennsylvania Health System and validated using data from Beth Israel Deaconess Medical Center to provide clinicians with recommendations related to electrolyte replacement. This approach was estimated to reduce magnesium and potassium replacement events by up to 60%, as well as a decrease in IV potassium replacement by 75% and oral replacement by 50%.

Few studies evaluated the burden associated with electrolyte overuse. In a study involving more than 300,000 visits across multiple ICUs from three hospitals, the estimated cost of electrolyte repletion administered within the normal reference range was approximately $1.25 million (19). This estimate included medication costs as well as the time-related costs of pharmacists, physicians, and nurses based on hourly wages. In the study by Prasad et al (27), implementation of an artificial intelligence–based reinforcement learning approach was projected to reduce medication costs during 5 years from $62,000 to $20,500. When incorporating laboratory, medication, and healthcare personnel costs, the total projected expenditures decreased from $519,000 to $156,000. In our study, we did not specifically assess cost savings associated with the Green-Lyte project but evaluated the environmental impact and pharmacy preparation workload. We demonstrated a 61.8% reduction in waste and a 63.2% reduction in material-associated carbon footprint, along with a 38.5% reduction in pharmacy preparation time.

This study provides evidence on the impact of optimizing the use of electrolyte replacement on drug utilization and the environmental burden. Nevertheless, several limitations should be acknowledged. First, this was a single-center, retrospective study, which is subject to the inherent limitations of this study design and may limit the generalizability of the findings. The study was conducted in a mixed medical-surgical oncology ICU, where the patient population and practice patterns may differ from those in general medical, surgical, or cardiac SICUs. In particular, the feasibility of oral electrolyte replacement may be lower in populations with a higher proportion of patients who are nil per os or managed under postoperative protocols that restrict enteral administration. Therefore, the magnitude of benefit observed with the Green-Lyte project may not be directly transferable to institutions with different ICU case mixes and practice patterns. In addition, given the retrospective nature of the study, we relied on clinical documentation to determine the rationale for electrolyte replacement and route of administration when these did not comply with the Green-Lyte criteria; however, it is possible that valid clinical justification existed but was not documented. Furthermore, the material-associated carbon footprint was estimated using published emission factors rather than direct environmental measurements. We also did not account for variations in laboratory follow-up monitoring, which may differ based on the electrolyte formulation used and may have an impact on waste generation, carbon footprint, and workload. Finally, we did not evaluate clinical outcomes associated with the Green-Lyte initiative. Therefore, although electrolyte utilization decreased and replacement practices became more concordant with the Green-Lyte criteria, we cannot determine whether these changes had an impact on patient safety, electrolyte-related adverse events, or treatment effectiveness.

## CONCLUSIONS

The Green-Lyte initiative was associated with optimized prescribing of electrolyte replacement and resulted in a decrease in waste, material-associated carbon footprint, and pharmacy workload. Future research should explore the implementation of this project in other critical care settings and to evaluate the clinical impact of such project.

## Supplementary Material
